# Collagen-Laponite Nanoclay Hydrogels for Tumor Spheroid Growth

**DOI:** 10.1021/acs.biomac.3c00257

**Published:** 2023-05-30

**Authors:** Pilar Alamán-Díez, Carlos Borau, Pedro Enrique Guerrero, Hippolyte Amaveda, Mario Mora, José María Fraile, Elena García-Gareta, José Manuel García-Aznar, María Ángeles Pérez

**Affiliations:** † Multiscale in Mechanical and Biological Engineering, Aragón Institute of Engineering Research (I3A) & Aragón Institute of Healthcare Research (IIS Aragón), Department of Mechanical Engineering, University of Zaragoza, Zaragoza, 50018, Spain; ‡ Aragon Institute of Nanoscience and Materials (INMA), University of Zaragoza & CSIC, Zaragoza, Aragon 50018, Spain; § Institute of Chemical synthesis and Homogeneous Catalysis (ISQCH), University of Zaragoza & CSIC, Zaragoza, Aragon 50009, Spain; ∥ Division of Biomaterials and Tissue Engineering, UCL Eastman Dental Institute, University College London, London, WC1E 6BT, United Kingdom

## Abstract

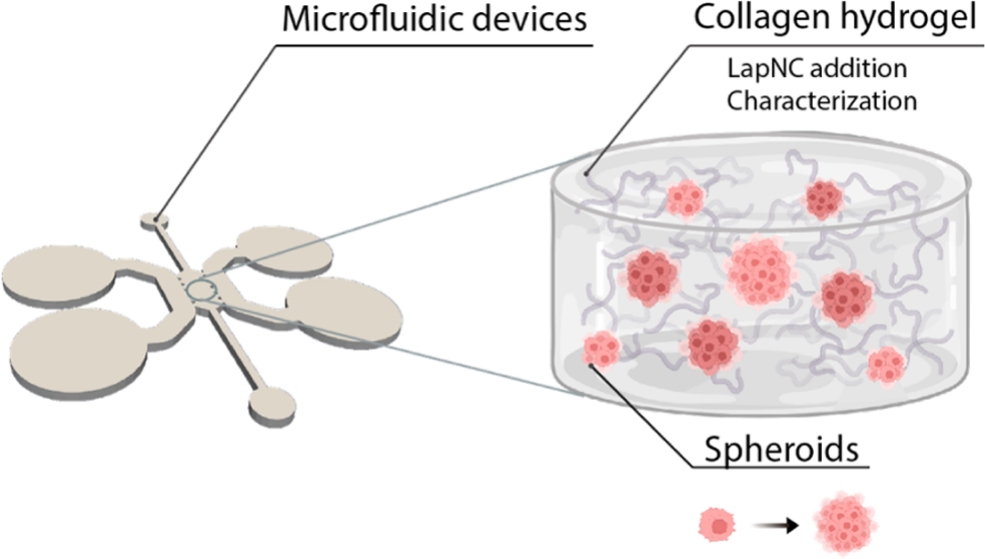

The extracellular matrix (ECM) plays an important regulatory role in the development and progression of tumoral tissue. Its functions and properties are crucial in determining tumor cell behavior such as invasion, migration, and malignancy development. Our study explores the role of collagen type I in cancer development and spread using engineered tumor models like multicellular spheroids grown in collagen-based hydrogels to simulate early tumor formation. We employ microfluidic techniques to test the hypothesis that (i) adding Laponite nanoclay to collagen hydrogels modifies mechanical and rheological properties and (ii) changing the stiffness of the collagen microenvironment affects tumor spheroid growth. Our findings support our theories and suggest the use of ECM components and engineered tumor models in cancer research, offering a biocompatible and biomimetic method to tailor the mechanical properties of conventional collagen hydrogels.

## 1 Introduction

As the most studied disease, cancer still remains a leading cause of death worldwide. Therefore, further research is still crucial to improve the prevention, detection, and treatment of all cancer types. Extracellular matrix (ECM) components are known to play significant regulatory roles in the tumoral tissue. The functions of the ECM and its intrinsic features are key in cancer tissue by, for example, triggering tumor cell behaviors and invasion,^1^ cell migration,^2^ and malignancy development.^3^

The importance of the tumor microenvironment has been recognized in recent years,^4^ as the interaction between the biochemical-physical cues of the tumor and its surroundings determine cancer pathway signaling. Besides, this microenvironment is crucial to understand tumor growth mechanisms and, therefore, is one of the main parameters involved in cancer treatment research.^5,6^ Collagen type I is the main component of the tumoral ECM, being a natural scaffold of the cancer cell microenvironment.^7^ Thus, changes in the amount of collagen in the host tissue could play a significant role in promoting the survival and growth of distant metastases and may support some zone-specific tumor spread.^8^

Engineered tumor models have proved to be useful tools in cancer research.^9^ To mimic the morphological and functional features of *in vivo* cancer tissue, multicellular tumor spheroids are grown and used as an *in vitro* model to mimic the first stages of tumor formation *in vivo*.^10^ Collagen-based hydrogels are widely used in tissue engineering to mimic the 3D physiological microenvironment.^11^ Additionally, collagen allows the fabrication of matrices with different mechanical properties based on their inherent composition and preparation procedures.^12^

Microfluidics enables miniaturization of early staged tumor growth.^13,14^ The use of microfluidics in tumoral spheroid research constrains the system to a submillimetric scale, obtaining a large reduction in cell number requirements, reagent volumes, wastes, and work space. Besides, it allows more control over the microenvironment and promotion of nutrient and biochemical diffusion through the hydrogel. Thus, cellular cultures embedded in collagen-based hydrogels in microdevices provide cells with a 3D environment that can better mimic physiological conditions compared to a conventional 2D monolayer culture, while taking advantage of the aforementioned strengths of microfluidics.

**Figure 1:**
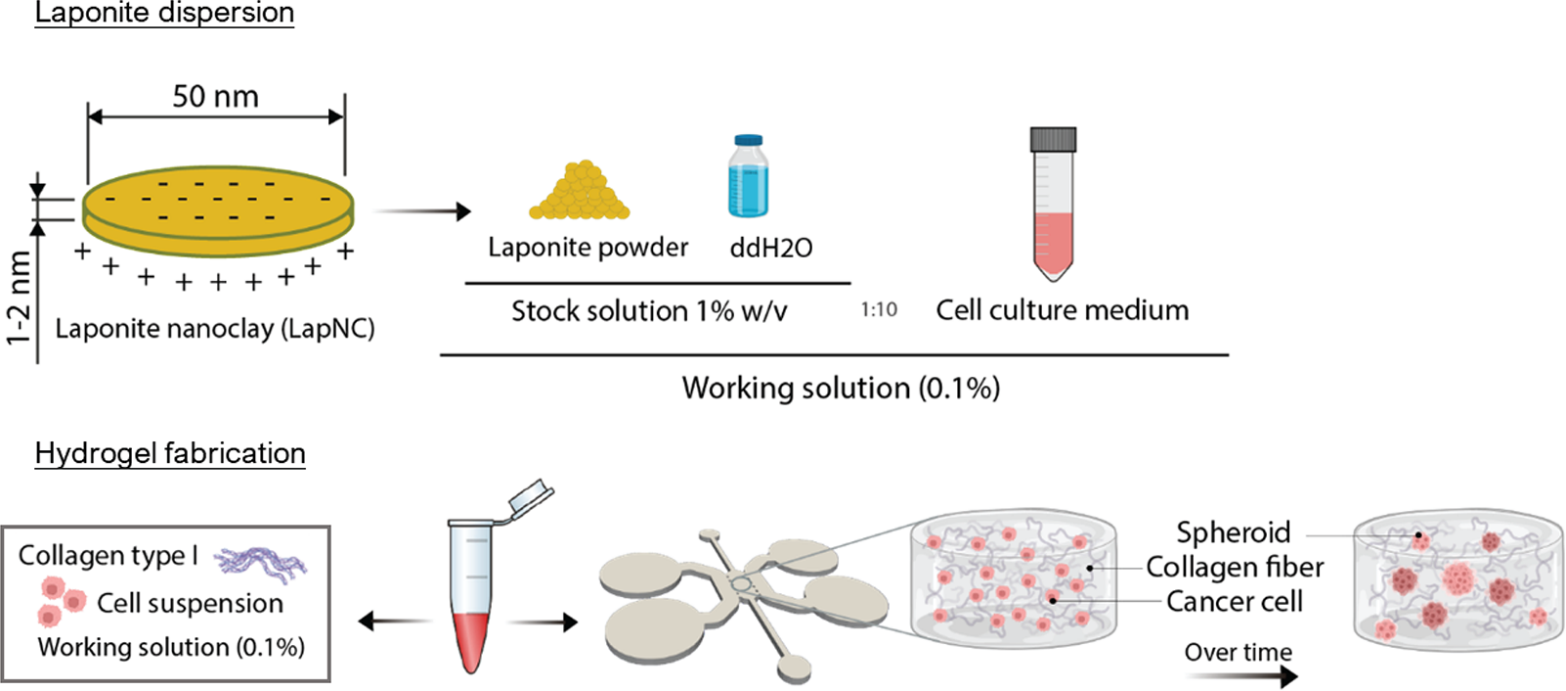
Experimental setup: Laponite nanoclay was supplied in powder form. It was diluted at 1% w:v in Milli-Q water and, subsequently, in culture media at 1/10 proportion. This working solution was used to fabricate the supplemented collagen hydrogels at different collagen concentrations. 3D tumor cell cultures were incubated in microfluidics, and the spheroids were formed from single-cell proliferation and cell–cell contact. The hydrogels were characterized by scanning electron microscopy, and in terms of swelling ratio and rheology gelled in wells. Additionally, permeability was characterized inside the microfluidic platform.

Laponite nanoclay (LapNC) is a layered synthetic silicate composed of inorganic salts. These nanoclays are characterized by the empirical formula:  (BYK Additives Ltd., UK). Gaharwar et al. thoroughly reviewed the biomedical applications of LapNC, among others: drug delivery, tissue engineering, imaging, cell adhesion and proliferation, and biosensors.^15^ Their hydrophilic characteristics and large surface area (>350 m^2^/g)^16^ facilitate physical interactions such as ionic interactions^17^ or hydrogen bonds^18^ with different molecules and proteins. Therefore, they have been widely investigated in the fields of regenerative medicine, additive manufacturing, and drug delivery. Individual clays have nanodisk-shaped geometry of 20–50 nm diameter and 1–2 nm thickness (Figure 1). Owing to its composition and size, the nanoclay exhibits a dual charge distribution: negative on the surface and positive along the edges. This property makes nanoclay dilution in liquid media a unique gel-like *house-of-cards* structure which can be implemented in different materials and biomaterials to enhance structure, mechanical properties, and general characteristics.^19^

This work was built upon two hypotheses. First, the addition of LapNC to collagen hydrogels would enhance the mechanical properties without collapsing the collagen matrix or forming clay aggregations. This could involve the possibility of customizing the mechanical and architectural properties of the tumoral *in vitro* ECM. Second, LapNC addition in the 3D collagen microenvironment would affect tumor spheroid growth. In such case, the outcomes from our study would demonstrate that collagen-LapNC hydrogels may provide a new 3D cancer culture system that mimics key aspects of the physiological tumor microenvironment.

The main goal of our study is to provide a new biocompatible and biomimetic method to easily tailor the mechanical properties of convectional collagen-based hydrogels. For this purpose, hydrogels with different collagen concentrations were modified with LapNC and characterized in terms of physical and rheological properties. Finally, those matrices were used to study the homotypic spheroid growth over time. Neuroblastoma spheroid growth was studied according to its size and shape over time under the different microenvironments inside the microfluidic device. To reinforce the conclusions of these experiments, other tumoral cell lines were tested: human epithelial lung and pancreas carcinoma lines. Even though the study of an *in vitro* cancer model is out of the scope of this work, to the best of our knowledge, this is the first report on the use of LapNC to promote tumoral spheroid growth from a single cell.

## 2 Materials and Methods

### 2.1 Experimental Setup

LapNC was used to enhance mechanical properties of collagen-based hydrogels at three different collagen concentrations (2.5, 4, and 6 mg/mL, respectively). Supplemented and nonsupplemented control hydrogels were fabricated for all the procedures herein explained. A single channel microfluidic device was used as a culture system to conduct the 15 day 3D cell culture incubation, which allows live tracking microscopy. The experimental setup is summarized in Figure 1.

### 2.2 Laponite Dispersion

LapNC (BYK Additives Ltd., UK) powder was 1% w:v diluted in Milli-Q water. This solution was subsequently dispersed in culture medium 1/10 to be applicable for cell culture, making a 0.1% w:v LapNC concentration in medium. The concentrations to prepare stock solution and the working solution were optimized to avoid clay precipitates and collagen collapse. It has already been reported that LapNC are totally cytocompatible at doses <1 mg/mL.^20,21^ Thus, the LapNC concentration used in this work did not hinder cellular viability.

### 2.3 TEM Inspection

**Figure 2:**
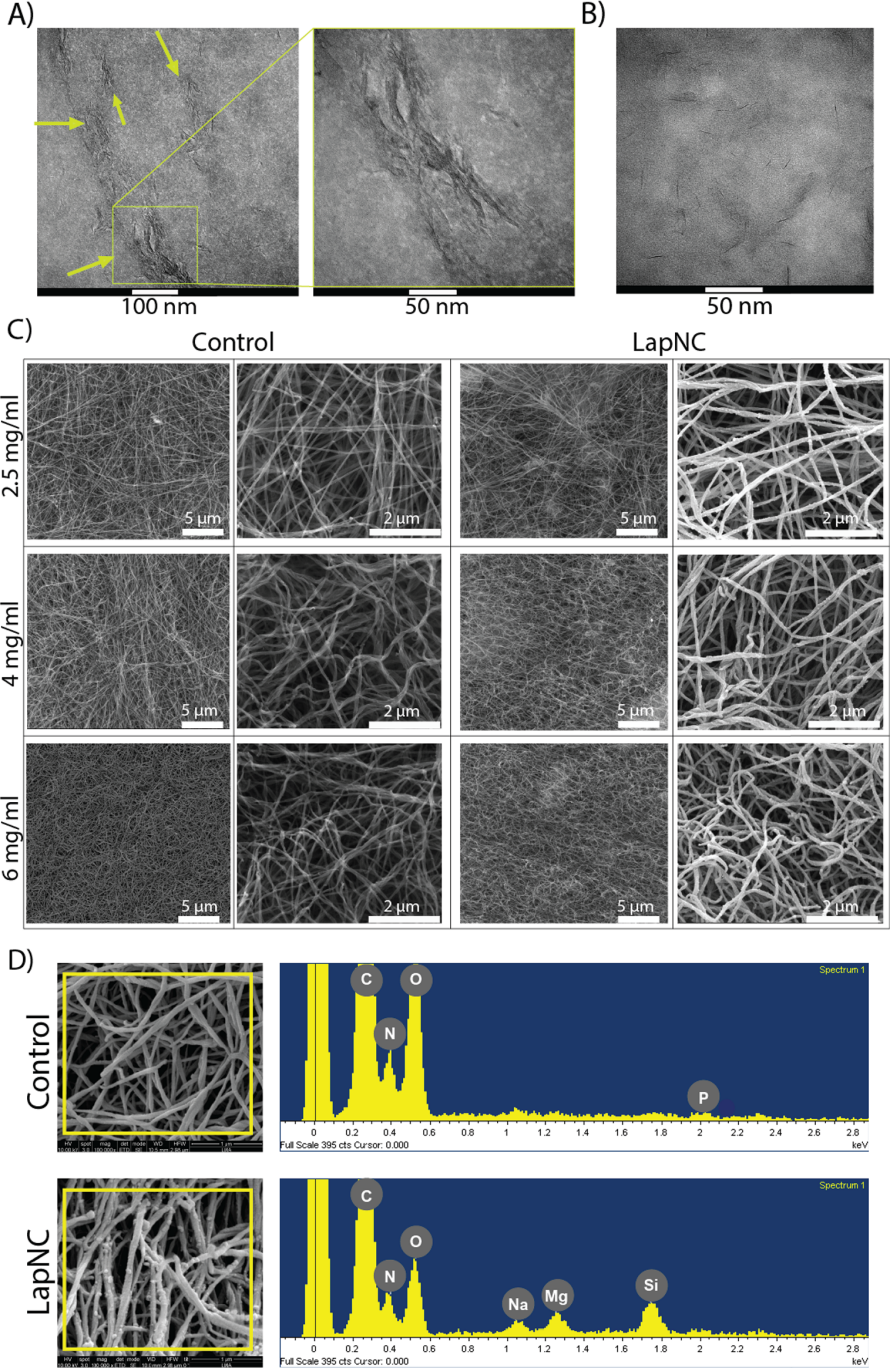
(A) TEM images of 1% LapNC stock solution and (B) 0.1% LapNC working solution. (C) Representative SEM images at at 25,000× and 50,000× magnification of the collagen hydrogels (2.5, 4, and 6 mg/mL) with/without LapNC. (D) Representative EDX report figures at 100,000× of a 6 mg/mL collagen hydrogel with/without LapNC.

Transmission electron microscopy (TEM) was used to image the Laponite dilutions and check homogeneous LapNC dispersion (Figure 2A and B). A 20 μL drop of each sample was poured on a freshly glow-discharged (30 s, 15 mA) carbon-coated 200-mesh copper grid (Agar Scientific Supplies, UK). Then, the grids were incubated for 2 min, and the excess was removed by contacting the grid edge with filter paper and allowed to air-dry. In order to check the size and morphology of nanoclays, the observation was conducted using Tecnai T20 (FEI, OR, US) microscope at 200 kV (Laboratory of Advanced Microscopy (LMA), University of Zaragoza).

### 2.4 Hydrogel Fabrication

Collagen hydrogels, with a final collagen concentration of 2.5, 4, and 6 mg/mL, were prepared by diluting in an ice bath collagen type I solution (Rat Tail, stock 10.8 mg/mL, Corning, NY, US) in Dulbecco’s Modified Eagle Medium (DMEM, 4.5 g/L glucose, Thermo Fisher Scientific, MA, US), 10× Dulbecco’s phosphate buffered saline (DPBS), and 0.5 M NaOH (both from Sigma-Aldrich, Germany) to adjust the pH to 7.4–7.6. Supplemented hydrogels were fabricated diluting the collagen in 0.1% LapNC-DMEM (working solution).

For characterization assays, 50 μL drops of the hydrogels were laid in wells (96 well plate). To conduct permeability and spheroid growth tracking, the hydrogels were gently pipetted inside the central culture chamber of a microfluidic device (see subsection 2.5.4, and Figure 1). After placement in the respective container, collagen solutions gelled for 20 min in a humid chamber at 37 °C. The temperature and the pH conditions mentioned above induced a self-assembled gelation process of the collagen hydrogels, where collagen fibers are physically cross-linked.^22^ During the gelation process, collagen fibers create an interpenetrating polymer network embedding the resuspended cells.^23^

### 2.5 Hydrogel Characterization

#### 2.5.1 SEM Inspection and EDX

First, samples were fixed in 4% paraformaldehyde for 15 min. Then, they were rinsed three times in phosphate buffered saline (PBS) for 5 min. After the fixation steps, the samples were subjected to sequential dehydration in graded series of ethanol. Subsequently, they carried a total water adsorption procedure by submitting them to a critical point drying stage (Leica EM CPD300 Critical Point Dryer, Germany). Finally, the samples were coated with a 20 nm carbon film before they were examined by scanning electron microscopy (SEM) and energy dispersive X-ray spectroscopy (EDX). Then, samples were visualized at 10 kV and 3 spot size, using a field emission SEM Inspect F50 (FEI, OR, US) in an energy range between 0 and 30 keV. To confirm the chemical composition of the samples, EDX was also using the Inspect F50 at 20 kV and 4 spot size (LMA, University of Zaragoza).

#### 2.5.2 Rheology

The gelation process of the hydrogels has been analyzed by means of linear oscillatory rheology using a control stress HAAKE RheoStress 1 (Thermo Fisher Scientific, Waltham, MA, US). The rheometer was connected to an external thermostatic bath. The measurements were carried out using a cone–plate sensor with a 35 mm radius and 1° angle. Once the hydrogel was prepared, it was placed in the lower plate at 20 °C. Then the upper cone was lowered to the measurement position, and the temperature is gradually raised to 37 °C. Once the sample has been equilibrated at this temperature, the hydrogel gelation was analyzed by applying a very low stress of γ = 0.45 Pa at a frequency of 0.1 Hz with a cyclic strain of 0.5% amplitude, which guarantees a torque of 5 μNm and a linear viscoelastic regime. To mitigate the evaporation of water from the hydrogel, a liquid trap was used. Storage moduli (*G*′) were recorded as a function of time using HAAKE Rheowin software (Job and Data Manager). Three samples were used for each test condition.

#### 2.5.3 Swelling Ratio

The swelling ratio (SR) of the different hydrogels was measured from the dry mass (*M*_d_) and wet mass (*M*_w_) of the hydrogels following eq 1. *M*_d_ was obtained by weighting lyophilized hydrogels (RADWAG-MYA5.4Y microbalance, 1 μg readability). *M*_w_ was acquired after immersing the sample into 500 μL of distilled water for 10 min at room temperature.
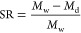
1

#### 2.5.4 Hydrogel Permeability

The resistance to flow exerted by the different hydrogels within the microdevices was measured by exposing them to a pressure gradient. Permeability was subsequently quantified by using Darcy’s Law. Microfluidic devices were fabricated as detailed in subsection 2.6.1, and the hydrogel was also prepared according to the procedure in subsection 2.4. The hydrogel was introduced in the central channel, and four empty columns were coupled in the medium ports of the device. The water inner columns were filled with PBS up to a known height, creating a pressure gradient through the hydrogel. Equation 2 provided the time-dependent relation with pressure and the material (constant *c*). Afterward, this constant was used to determine the Darcy’s permeability *K* in *m*^2^ according the eq 3.
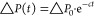
2
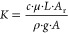
3with μ and ρ being the viscosity and the density of the fluid, respectively, *L* the length of the gel through which the pressure drop was established, *A*_r_ the area of the media reservoirs, *g* the gravitational acceleration, and *A* the cross sectional area to flow. It has been reported that the permeability decreases as the collagen concentration increases.^24^ Besides, we hypothesized that LapNC may enhance this effect due to their nanoscale biochemical and morphological properties.

### 2.6 Tumoral Spheroid Culture in Microfluidics

#### 2.6.1 Microdevices Fabrication

Microfluidic devices were fabricated in poly(dimethysiloxane) (PDMS) by soft lithography, following the methodology described by Shin et al.^25^ A commercial product was used to produce the silicone elastomer (Sylgard 184 Silicone Elastomer Kit, Dow Chemical, Germany), which comprises a polymeric base and a silicone resin solution as curing agent. The two liquid parts were mixed in a 10:1 (base:curing agent) ratio and poured in a master made of an epoxy (SU-8, Micro Resist Technology GmbH, Germany) where the microengineered geometry was patterned with a photolithography technique (Aragón Nanoscience and Materials Institute). The geometry consists of one single culture chamber which is connected with two reservoir channels for the culture hydration. Figure 1 renders the inner geometry of the microfluidic device.

The geometry masters were then placed in a vacuum desiccator for 1 h, to remove air bubbles in the PDMS solution, and kept in a dry oven overnight to cure the mixture. Later, dermal biopsy punches were used to create the reservoirs for cell culture media and the hydrogel inlets to the culture chamber. PDMS devices followed a wet and a dry autoclave cycle before being bonded to a 35 mm glass (Ibidi, Germany) by plasma treatment (PDC-32G Basic Plasma Cleaner, Harrick Plasma, NY, US) under vacuum conditions. They were then coated with PDL (poly-d-lysine; 1 mg/mL in PBS; Sigma-Aldrich, Germany) and washed after 4 h to enhance matrix adhesion. Before use, the devices were left in a dry oven at 80 °C for 48 h to restore the hydrophobicity of the bonded surfaces.^25^

#### 2.6.2 3D-Cell Seeding

Tumoral cells were cultured (2D) under standard conditions (5% CO_2_, 37 °C) up to 70–80% confluence in their regular expansion medium: DMEM high glucose (4.5 g/L) supplemented with 10% fetal bovine serum (FBS, Thermo Fisher Scientific, MA, US), 100 U/mL penicillin, 100 μg/mL streptomycin, and 2 mM l-glutamine (all from Lonza, Switzerland). For cell expansion, cultures were washed with PBS, detached with TrypLE Express (Thermo Fisher Scientific, MA, US) and plated in T25 cell culture flasks at a density of 15,000 cells/cm^2^.

Cell laden collagen hydrogels (both supplemented and nonsupplemented) were fabricated as detailed above (subsection 2.4), including cell content. The tumoral cells were suspended in expansion medium at a concentration of 150,000 cells/mL in the collagen solution. After gently pipetting the solution into the culture chamber, it gelled for 20 min in a humid chamber at 37 °C. Once the hydrogel is gelled, the culture is hydrated through the media ports with regular DMEM. This media was exchanged every 48 h to ensure nutrients delivery to the cultures.

Over time, spherical cluster formation from singe-cell proliferation in the culture chamber (Figure 1) was expected in a stiffness-dependent manner. These spheroid growth were tracked for three different cell lines: Neuroblastoma (PACA), human lung epithelial carcinoma (A549), and human pancreatic ductal carcinoma (PANC-1). Moreover, PACA cells were used to compare the initial migration and cluster formation in the softest hydrogel.

### 2.7 Multicellular Structures Morphology Tracking

#### 2.7.1 Imaging Overtime: Spheroids Growth Quantification

Samples were imaged in brightfield at 4× magnification every 48 h up to 15 days of culture with an optical microscope (Leica DM IL LED, Germany). The images were analyzed with a custom semi-automatic segmentation algorithm based on active contours,^26^ which was developed in Matlab (Mathworks, Natick, CA, US), to quantify the spheroid area over time. Figure S3 in Supporting Information shows the shows a screenshot of the app while segmenting a particular image.

Both qualitative (microscopy pictures) and quantitative (area measurement) data were obtained for the 3 cell lines. However, only PACA data was shown in the main text as representative results. Figure S2 in Supporting Information contains the information corresponding to PANC-1 and A549 lines, respectively.

#### 2.7.2 Cell Migration

For studying 3D cell migration, collagen type-I hydrogels were fabricated following the methodology detailed in subsection 2.4 with minor modifications: PACA cells were mixed with 2.5 mg/mL collagen type-I (with/without 0.1% LapNC) at a final concentration of 2 × 10^5^ cells/mL. After filling the microdevice central chamber, the devices were placed in humidified chambers in a CO_2_ incubator to allow the collagen to gel at 37 °C for 20 min. To promote cell migration, cells were serum starved without fetal calf serum. After 24 h, the medium was replaced with DMEM supplemented with 10% FBS. Then, time-lapse imaging of the devices was carried out with a D-Eclipse Ti Microscope (Nikon, Japan) with a 10× objective, acquiring contrast images every 20 min for 24 h to study cell migration at 37 °C in a humidified atmosphere and 5% CO_2_. The central part of the device along the *Z*-axis was selected as focal plane to ensure that the tracked cells were embedded within the 3D network. Thus, out-of-focus cells were not quantified to minimize artifacts resulting from two-dimensional migration.

At least 50 cells for each device were manually tracked along the 73 time-frames with the Manual Tracking plugin from ImageJ (National Institutes of Health, MD, US). Subsequently, individual cell migration was analyzed with a custom code developed in Matlab^27^ used in previous works.^8,28,29^ These trajectories were used to extract the mean *V*_mean_ and effective *V*_eff_ velocities, defined as the average instantaneous speed including all time steps, and the speed calculated using only the initial and final positions, respectively. The mean square displacement (MSD) curve of each trajectory was used to determine the global diffusion coefficient (*D*) as a measure of cell motility and migration persistence.^30^ Additionally, MSD curves were used to fit a power law (MSD(*t*) = γ × *t*^α^) to determine the kind of motion (α < 1 for confined, α = 1 for Brownian or purely diffusive, and α > 1 for directed motion, respectively).

#### 2.7.3 Nuclei Tracking

For live nuclei visualization at the beginning of the cluster formation, Neuroblastoma spheroids with PACA-Green Fluorescent Protein (GFP) (transfected with a plasmid containing histone 2B fused to GFP) were imaged (at days 1 and 3) with the Lattice Lightsheet 7 microscope (ZEISS, Germany) in a manner that is compatible with long-term fluorescent time-lapse imaging. Images were acquired with a 40× water immersion objective lens using both heating and gas incubation system (Ibidi, Germany), to achieve a humidified atmosphere at 37 °C with 5% CO_2_. Fixed and stained spheroids were also imaged, processed (Deconvolution and Deskew), and 3D projected using Zen 3.5 Blue software (ZEISS, Germany).

### 2.8 Statistics

Every condition was tested in triplicate. After data normality assessment via quantile–quantile and normal probability plots, analysis of variance (ANOVA) followed by post hoc Tukey–Kramer tests were performed to determine statistical significance among the studied continuous variables in the different conditions. Non-parametric (Kruskal–Wallis) tests followed by posthoc Tukey–Kramer tests were used instead when data distribution was not normal. Results presented in box plots show median, quartiles, maximum and minimum values, and outliers.

## 3 Results and Discussion

### 3.1 LapNC Dispersion and Hydrogel Microstructure

Figure 2 shows the Laponite dispersion. The stock dilution in water at 1% w:v (Figure 2A) showed the presence of platelets of grouped clays, as reported before.^31^ The results of directly mixing the stock solution with collagen involved clay and collagen fiber aggregation. Thus, the stock solution was subsequently diluted in culture medium at 0.1% w:v as commented in subsection 2.2. This working solution, LapNC in DMEM (Figure 2B), was the one used to fabricate the hydrogels (subsection 2.4). TEM images evidenced the homogeneous dispersion of LapNC in DMEM at the selected concentration.

Figure 2C shows representative SEM images of every hydrogel at different collagen concentrations with/without the addition of Laponite. A porous matrix was achieved for all the categories, with a wide range of pore sizes. The hydrogels were heterogeneous and fibrous networks with a large superficial area for cells to attach. The presence of nano- and micropatterns in the fabricated collagen matrix is very relevant as they appear in the natural ECM and cells are naturally *in vivo* in contact with both nano and microfeatures.^32^

The addition of LapNC at 0.1% did not raise any clay aggregation or collagen structure collapse, which were indeed observed in preliminary tests with more concentrated LapNC stock dispersions. Moreover, this addition of LapNC can be observed in Figure 2C as little speckles stuck along the collagen fibers. The nanodisk attachment to collagen fibers may be due to electrostatic interaction of LapNC with the protein side charged chains or because of the formation of hydrogen bonds.^17,18^ It was also qualitatively evidenced that the increase in the collagen concentration involved a denser matrix and a reduction in the fibrous pore sizes.

The presence of Laponite in the hydrogel was checked by EDX analysis. Figure 2D shows representative spectra from a gel with and without LapNC. With the formula , the appearance of magnesium (Mg), sodium (Na), and silicon (Si) ions in the LapNC spectra confirms the presence of the nanoclay. Lithium (Li) could not be observed possibly due to its low proportion.

In agreement with our results, Shi et al. created a Laponite supplemented collagen for a leather matrix fabrication. By using atomic force microscopy (AFM), they showed that the primary and conformational structures of collagen molecules are not disturbed by the introduction of Laponite,^33^ similar to our own. Other authors also investigated the molecular interactions occurring in LapNC addition to collagen-based biomaterials.^34^ They showed that the nanoclays bind onto the surface of collagen fibrils and cross-link with the collagen molecules through noncovalent interactions (such as hydrogen and ionic bonding).

### 3.2 Quantification of the Mechanical Properties

#### 3.2.1 Rheology Confirms the Enhancement of the Matrix Stiffness

**Figure 3:**
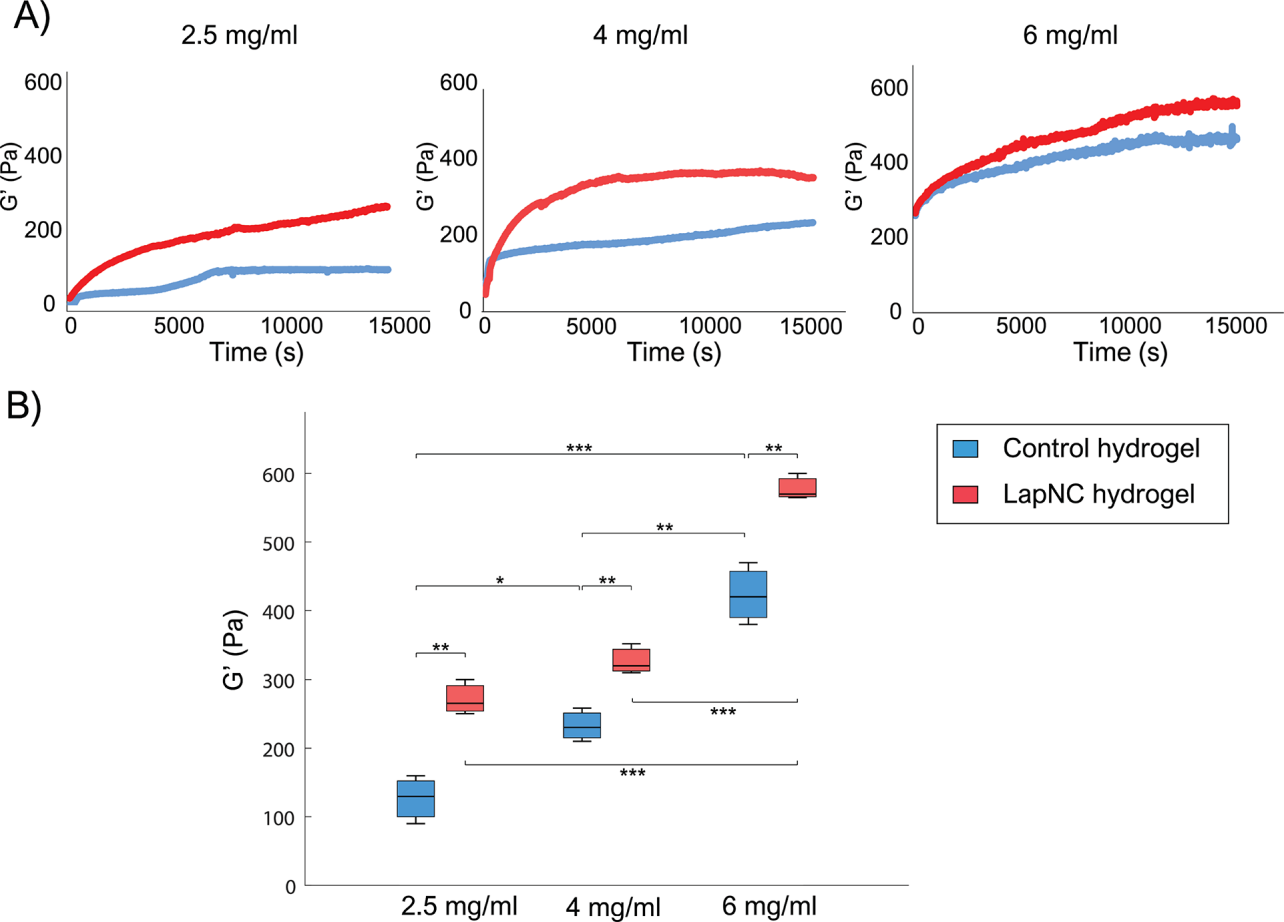
(A) Single representative gelation curves for each collagen concentration (2.5, 4, and 6 mg/mL). (B) Storage moduli (*G*′) after the hydrogels gelation. *n* = 3, * *p* < 0.05, ** *p* < 0.01, *** *p* < 0.005.

The enhancement of the mechanical properties of the hydrogels by the addition of nanoclay was confirmed by rheology. Storage modulus (*G*′) was measured during the gelation period of the hydrogel. Figure 3B plots the *G*′ values after the gelation period of the different hydrogels.

It can be observed that higher collagen concentration hydrogels involved a stiffer matrix, as could be expected.^35^ In addition, we observed significant differences (*p* < 0.01) between supplemented and nonsupplemented hydrogels for the three collagen hydrogel concentrations, which supports our initial hypothesis.

Interestingly, both LapNC supplemented 2.5 mg/mL and control 4 mg/mL, present similar storage modulus (as well as the LapNC supplemented 4 mg/mL and control 6 mg/mL pair). Therefore, it seems feasible to fabricate hydrogels with the same stiffness (same *G*′) but having different collagen concentration and, hence, different microstructure (Figure 2C). In agreement with our results, Li et al. showed the enhancement of mechanical properties of a 5 mg/mL cattle skin collagen hydrogel at different Laponite concentration addition. They showed that the elastic modulus, fracture stress, compression modulus, and rupture point of hydrogel treated by 10% LapNC were 695.2 Pa, 253.3 kPa, 13.5 kPa, and 76.7%, respectively, which were about 3.5, 5.5, 45.0, and 1.4 times larger than those of regular collagen-based hydrogel.^36^

This enhancement of mechanical properties due to the addition of nanoclays to the hydrogel could be used for different purposes. For instance, to promote osteogenic differentiation of mesenchymal stem cells *in vitro*, it has been reported that human bone marrow stem cells seeded onto surfaces coated with air-dried Laponite films (2.5 mg/mL) possess a higher osteogenic capacity at week 3.^21^ The supplemented collagen hydrogel reported here could be used to promote osteogenic differentiation of stem cells in a 3D matrix.^37^ Furthermore, the tumoral environment has been widely reported to be mechanically stiffer than healthy tissue,^38^ particularly in solid tumors. Therefore, given the mechanical characteristics of the tumoral microenvironment, the collagen-based hydrogel developed in this study may be a suitable tool for cancer research.

#### 3.2.2 Swelling Ratio Variation

**Figure 4:**
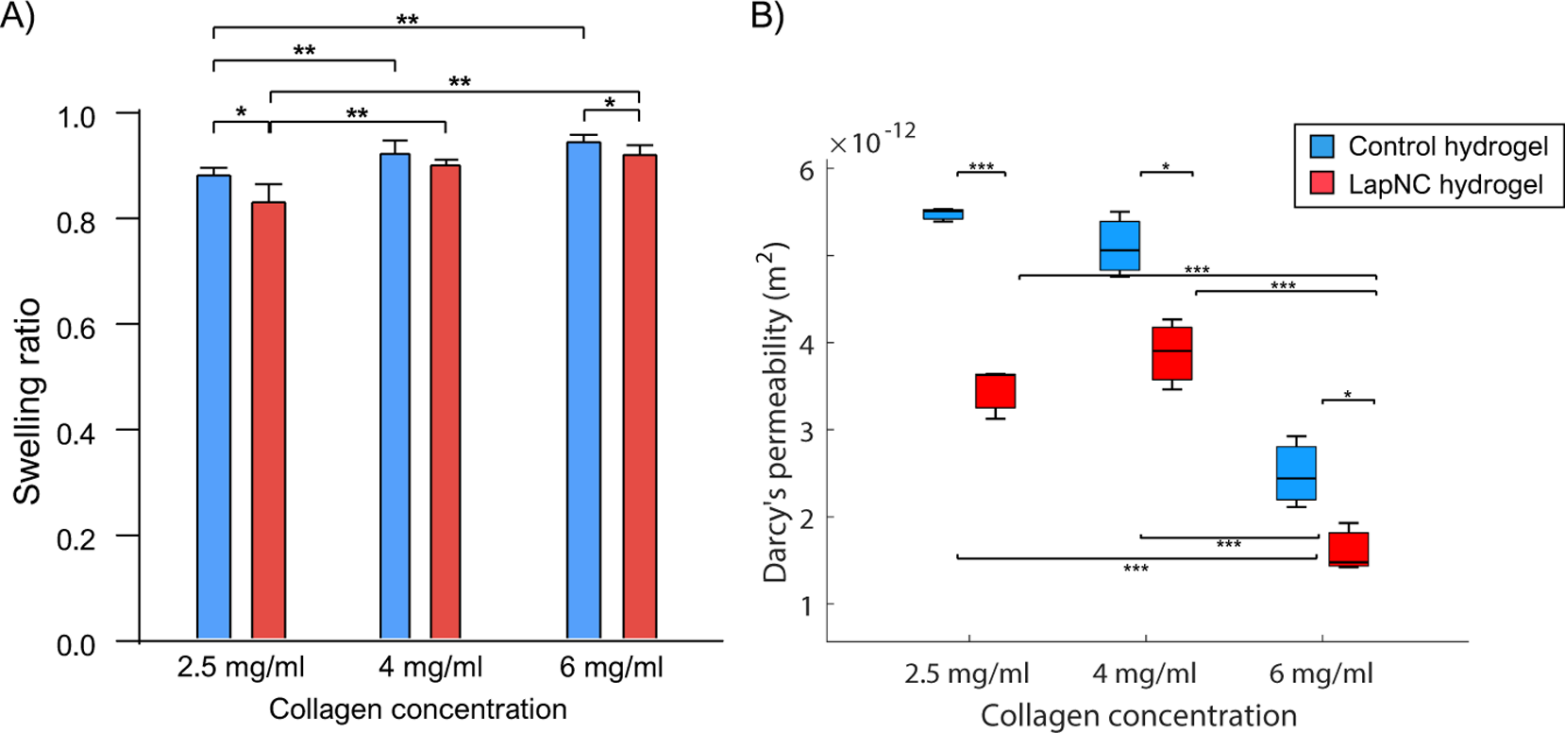
(A) Swelling ratios (mean ± SD) of the different hydrogels: 2.5 mg/mL, 4 and 6 mg/mL of collagen concentration, with (red) and without (blue) the addition of 0.1% Laponite solution. (B) Darcy’s permeability for collagen hydrogels, with (red) and without (blue) the addition of LapNC. Significance for * *p* < 0.05, ** *p* < 0.01, and *** *p* < 0.005.

SR value goes from 0 (no water adsorption) to 1 (total water adsorption). As can be seen in Figure 4A, a higher collagen concentration exhibited more water retention capacity, as can be expected.^39^ These results are in agreement with other published studies. For instance, Sawadkar et al. calculated the swelling ratio for collagen and elastin scaffolds, and they obtained 92.37% and 35.73%, respectively.^40^ This indicates that the solvent absorbing capacity of elastin scaffolds was weaker than collagen scaffolds, as it happened with low collagen concentration hydrogels.

According to results from the literature, we hypothesized that the addition of LapNC to the collagen matrix would decrease the hydrogel SR. As it can be seen in Figure 4A, LapNC supplemented hydrogels (2.5 and 6 mg/mL) presented significant differences. Farahnaky et al. showed the increase in a gelatin matrix SR with the addition of different % of clay nanocomposite (0, 2, 6, 10, 14, 18%).^41^ Additionally, Nair et al. conducted a mechanical behavior study of modified poly(vinyl alcohol)/laponite nanocomposite membranes. The authors evidenced that nanoclay membranes exhibited a decrease in swelling with an increase in laponite content.^42^ Aggregations of individual fibers by the presence of LapNC increased stability of polymer chains through molecule interactions and thereby reduces their water uptake capacity.^43,44^

#### 3.2.3 Permeability Reduction in Supplemented Hydrogels

Darcy’s permeability *K*[*m*^2^] was quantified thanks to the introduction of a water pressure gradient through the samples. Figure 4B shows the values of *K*. It can be observed that collagen concentration greatly affected the collagenous matrix permeability.

Additionally, LapNC addition also had a significant effect on the permeability regardless of the collagen concentration. As commented previously, LapNC present physical surface area above 350 m^2^/g.^45^ Thus, the LapNC supplemented hydrogel possessed more tortuous paths for a particle to migrate through the sample, further reducing the rate of the movement of water molecules between different phases.^46^

In agreement with our results, Kanmani et al. indicated that the incorporation of nanoclay was able to decrease water vapor permeability of the gelatin film mainly because of the presence of a tortuous pathway for water vapor diffusion caused by impermeable silicate layered nanoclay.^47^ Our experiments corroborated this, with the liquid column experimental setup used, getting a significant reduction in the permeability value with the addition of the nanoclays.

Busby et al. thoroughly characterized different concentration of collagen solutions assaying rat tail tendon collagen type I. In agreement with our results, they showed the relation of collagen concentration with the enhancement of mechanical properties and the SR, as well as the reduction of the permeability.^24^ A few previous studies have found that the addition of LapNC dispersion in the collagen matrix significantly modified those physical properties.^24,41,42,48^ However, none of them applied these matrices to cancer research. Regarding the relevance of the mechanical properties in the tumoral microenvironment, the achieved modification of the matrix properties could be useful in a culture system for studying *in vitro* cancer models. This outcome also highlights the flair of LapNC in cancer research, since tumoral tissue ECM has been shown to exhibit a lower permeability than the one of healthy tissue.^49^

### 3.3 Spheroid Growth Evolution Altered by the LapNC Addition

**Figure 5:**
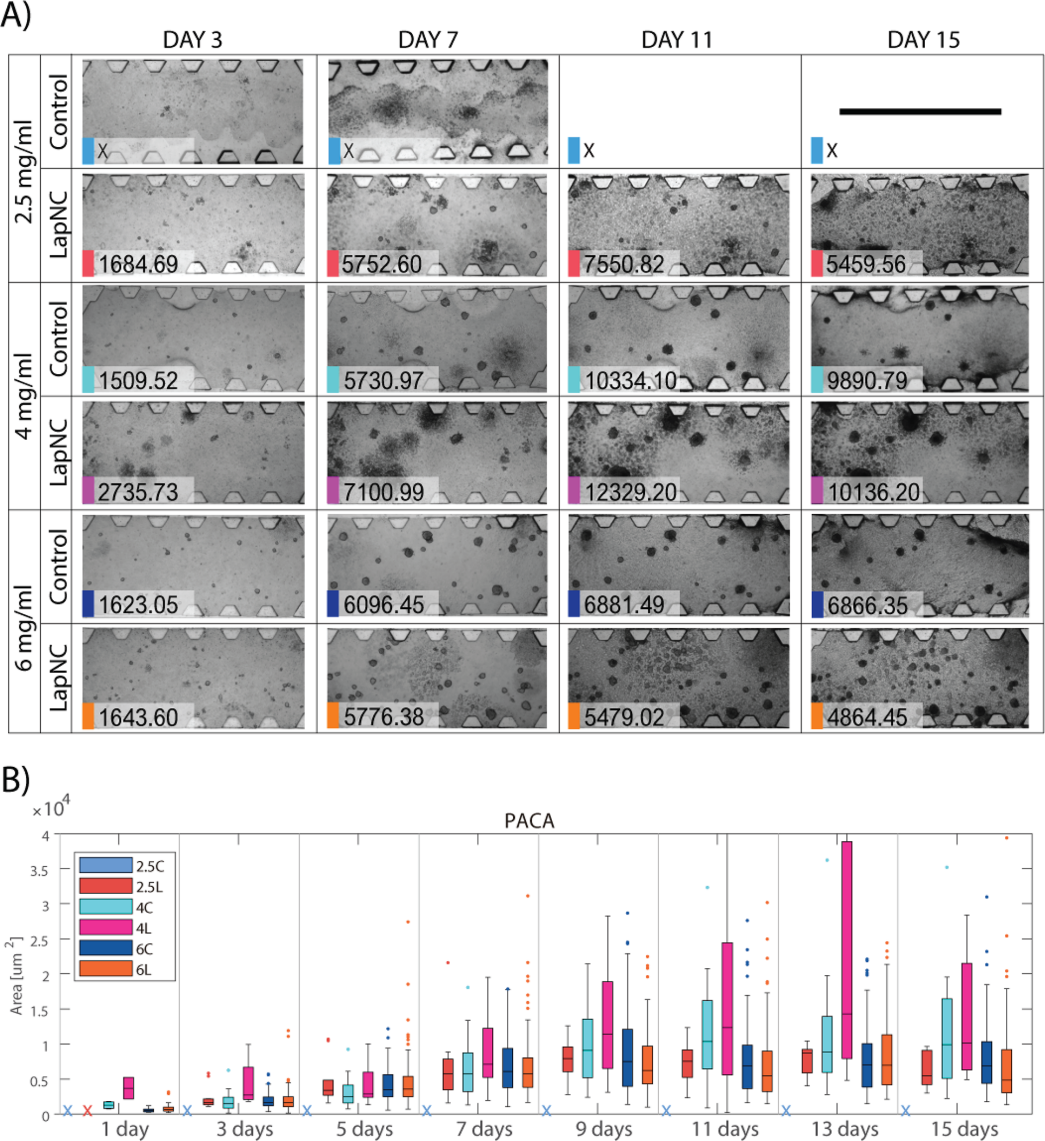
(A) Brightfield images of a representative PACA culture sample of each condition (collagen concentrations with/without LapNC) at 4 different time points. Each representative image is accompanied by the spheroid area median (μm^2^) in that condition. The symbol X denotes that the hydrogel collapsed at this time point or that no cells remained in 3D in this condition (total migration to the bottom surface). Scale bar in black 2 mm. (B) PACA cells spheroid area (μm^2^) distribution over time (shown as box plot): 3D cultures in different matrix: 2.5, 4, and 6 mg/mL of collagen; with and without the addition of LapNC. Conditions not able to form spheroids at specific time points are marked with colored crosses.

Cell cultures were imaged by brighfield tracking every 48 h up to 15 days of culture. Figure 5A qualitatively shows the evolution over time of PACA cells cultured at different conditions. It was possible to observe the creation of the neuroblastoma spheroids (day 15) from single-cell growth (day 1). In other methods to fabricate spheroids, such as hanging drop method or ultralow attachment culture substrates,^50^ spheroids are formed by the physical aggregation of a cell suspension. Unlike those methods, in our experiments, the collagen-based matrix properties (specially its architecture and stiffness) and its interaction with the cells were responsible for regulating spheroid growth.^8^

Over time, we generally observed that larger spheroids were formed in LapNC supplemented hydrogels. The differences between supplemented and nonsupplemented hydrogels are specially decisive in low collagen concentration: we saw that 2.5 mg/mL (without LapNC) is a not able to hold the PACA multicellular structure formation in 3D. This fact was probably due to the lack of stiffness (Figure 3) and the low collagen density in the matrix (Figure 2). Thus, 2.5 mg/mL hydrogel with no clays resulted in a detachment from the channel, cell movement to the glass bottom (2D) or temporary 3D culture with cell proliferation but not spheroid formation. However, LapNC supplemented 2.5 mg/mL hydrogel not only was stable up to 15 days in the device (Figure 5A) but was also able to hold small spheroid formation. On the other hand, we did not notice significant differences at first sight between the control 4 and 6 mg/mL collagen content.

Additionally, we quantified the spheroid growth over time via image analysis. Figure 5B shows the evolution of the spheroid area in the PACA cultures at every condition. We observed that increasing the collagen concentration (4 mg/mL over 2.5 mg/mL) involved larger emerging spheroids. We also confirmed quantitatively no significant differences between control 4 and 6 mg/mL matrix (*p* > 0.16 for all the time points). Moreover, no statistical differences in spheroid size were found for supplemented and nonsupplemented 6 mg/mL hydrogel (*p* ∼ 0.9 for all the time points) despite its variation of mechanical properties (Figure 3).

However, significant changes were observed for low collagen concentration hydrogels (2.5 mg/mL). This matrix with no LapNC exhibited the lowest mechanical properties (Figure 3) and, consequently, did not stand 15 days of culture. In fact, no PACA spheroids were observed for 2.5 mg/mL control at any time point (Figure 5A); thus, no boxes are shown in the figure. Either the collagen matrix collapsed or cells moved to 2D by gravity, rapidly proliferating in 2D over the glass. Thus, cell incubation longer than 7 days was not technically feasible. Nevertheless, LapNC supplemented 2.5 mg/mL collagen hydrogel presented a more stable cell culture platform. On one side, supplemented matrix allowed the incubation for longer periods due to a superior stability of the hydrogel. On the other side, as can be appreciated in Figure 5A second row and B 2.5L data, cells were able to form spheroids embedded in the supplemented matrix.

**Figure 6:**
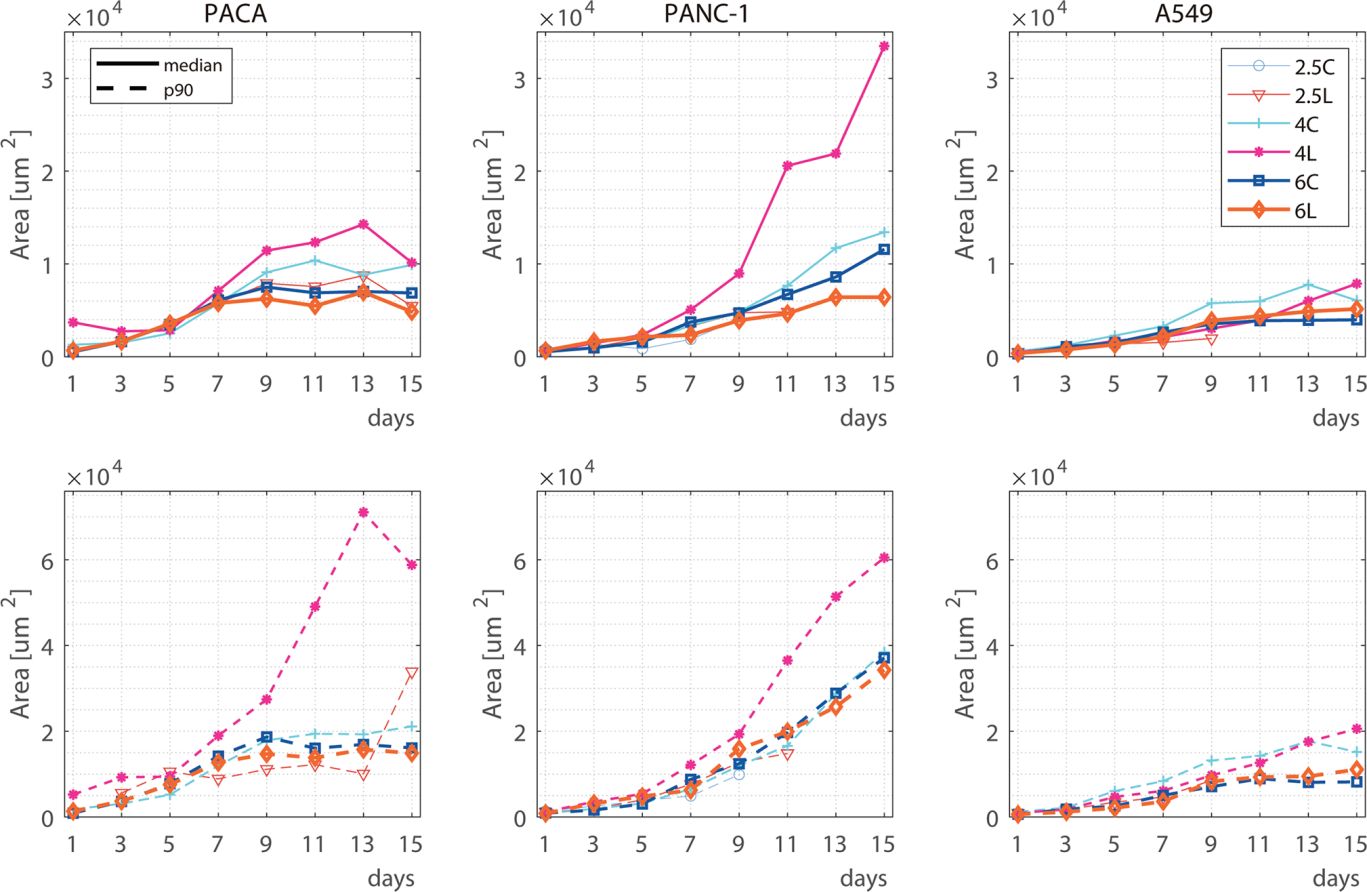
Evolution over time of the different cell lines at each condition: 2.5, 4, and 6 mg/mL of collagen with and without the addition of LapNC. Continuous lines show the median values, while dashed lines represent the 90% percentile.

Differences in spheroid growth at 4 mg/mL matrices, compared to softer ones, are easily appreciated. In fact, the enhancement of the mechanical properties of the matrix with LapNC lead to the largest spheroids for all the cell lines (Figure 6), highly evidenced from day 11 of culture on. This confirms the possibility to obtain large multicellular units embedded in a matrix not only by increasing collagen concentration but also by alternatively adding LapNC.

Regarding the highest collagen concentration matrix (6 mg/mL), no large differences in spheroid growth were observed comparing LapNC supplemented and nonsupplemented hydrogels despite the increase in *G*′ (Figure 3). Zanotelli et al. suggested that at high collagen concentrations, with superior mechanical properties, the proliferation capacity of cells was highly reduced, as incubated in that kind of microenvironment, cells would only possess low motility capacity.^51^ This might explain that there is a correlation between substrate stiffness and spheroid area up to a stiffness threshold where the matrix impedes cell proliferation to enlarge the spheroid.

Furthermore, matrices with comparable rigidity, such as 2.5L and 4C, as well as 4L and 6C, were investigated to compare cell behavior, as they exhibited no statistically significant differences in terms of storage modulus *G*′ (Figure 3B). Nevertheless, statistically significant differences were observed regarding the growth of spheroids among these matrices (Figure 5B), specially between 4L (pink) and 6C (dark blue) from day 11 on (*p* = 0.0302). The observed effect can be attributed to the distinct microarchitectures exhibited by each of these hydrogels, as evaluated through the permeability measurements (Figure 4B).

The results obtained for PACA cell lines are corroborated with the PANC-1 and A549 lines. The figures in the Supporting Information (S1 and S2, respectively) show both the qualitative and quantitative analysis of both lines tracking. Nevertheless, to show a full comparison, Figure 6A shows the spheroid area evolution for all collagen conditions and all cell lines. It is worth noting that new spheroids appeared at any given time within the gels and that we did not track them individually. This entails that spheroids at different stages of their evolution (and therefore different sizes) are present at the end of the experiments. To overcome this, the figure shows both the median area value and the percentile 90 (Figure 6B), which better represents the potential of each condition to produce large spheroids. Supporting Information Table S1 shows the numerical values at the final time of these median and p90 values for the three cell lines. As can be seen, the LapNC supplemented 4 mg/mL collagen hydrogel is the matrix which enables the largest spheroid formation for all cell lines, but especially for PACA and PANC-1. This, together with a higher median value over time, suggests that it is not a case of single spheroids growing large, but rather that all of them grow, overall, faster.

### 3.4 Cell Migration Reduction in Supplemented Matrix

**Figure 7:**
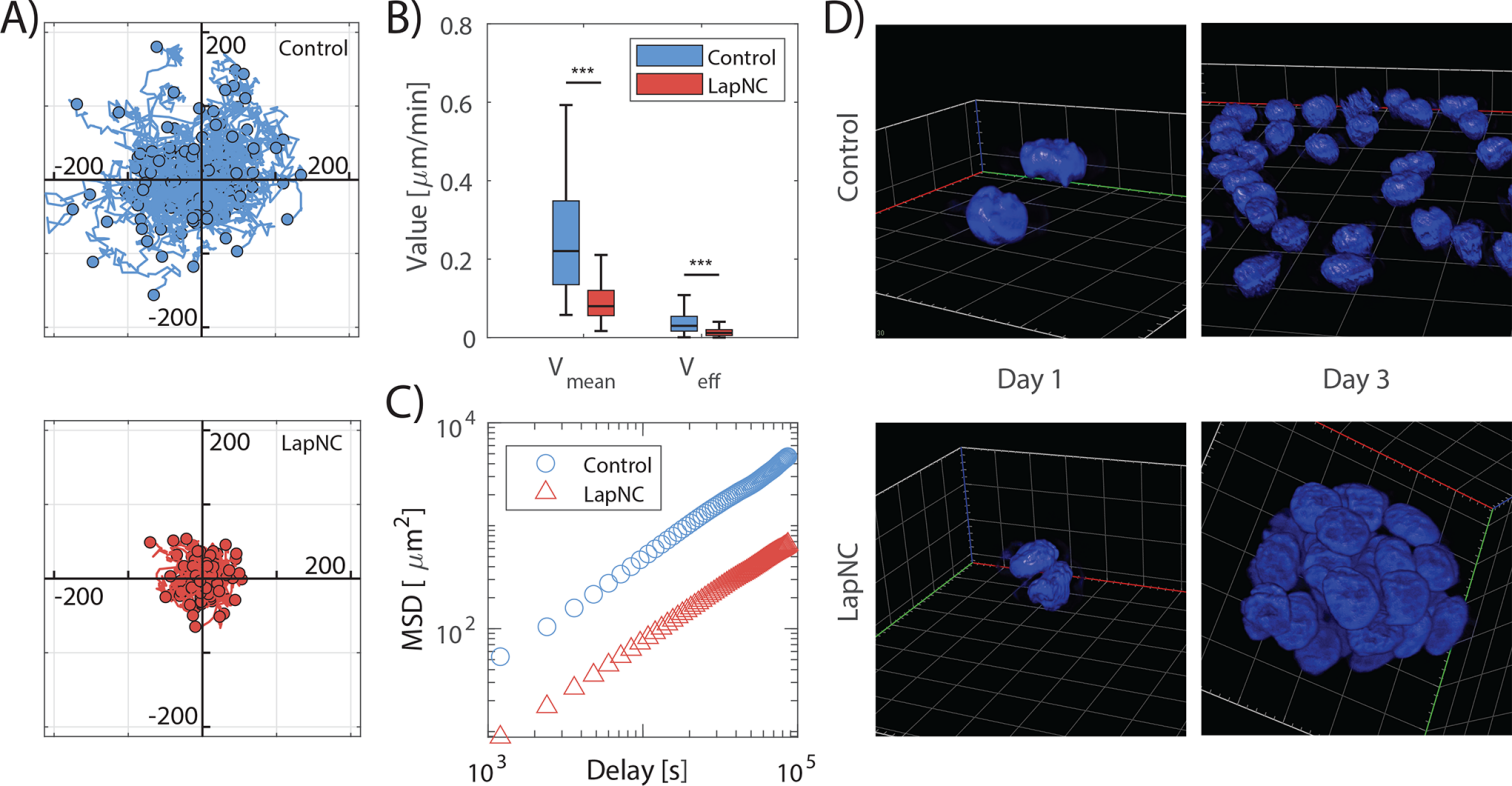
PACA cell migration and beginning of cluster formation within LapNC supplemented (red) and control (blue) 2.5 mg/mL collagen hydrogels: (A) Relative trajectories of cells collected from control and LapNC samples (60 events per sample, *n* = 3), *X* and *Y* axis in μm. (B) Values of *V*_mean_ (left) and *V*_eff_ (right) of the cell migration in both types of samples. Statistical significance * *p* < 0.05, ** *p* < 0.001. (C) Mean square displacement (MSD) of the tracked trajectories. Three devices were analyzed per condition used to study more than 150 cells individually. (D) 3D projection of representative PACA cells at day 1 and 3 of culture. Images were processed and 3D volume was projected with Zen 3.5 Blue software (ZEISS, Germany).

This analysis was only conducted in the soft hydrogel (2.5 mg/mL) because previous studies have shown that no relevant migration is observed in 4 and 6 mg/mL collagen hydrogels.^8^ In this work we observed that PACA cells migrated significantly slower when they were embedded within the 3D network with LapNC nanoclays. Both the mean (*V*_mean_) and effective (*V*_eff_) velocity (Figure 7B) were significantly decreased when adding LapNC.

The LapNC addition to the collagen hydrogels led to a marked decrease in the migrated distances of the cells as shown in Figure 7A where each of the relative cell trajectories are represented. Since the LapNC supplemented matrix is significantly stiffer (Figure 3), cells become more confined, which make migration more arduous, ultimately leading to shorter cell trajectories^8^ and lower speeds. This is further confirmed by the MSD curves (Figure 7C) where both LapNC and control conditions were found slightly subdiffusive (confined motion) but with a much lower diffusivity in the former (*D* = 0.11 μm^2^/min and α = 0.94) ± 0.27, as compared to control (*D* = 0.77 μm^2^/min, α = 0.93 ± 0.29).

The observed difference in motile capacity for supplemented and nonsupplemented matrix may be explained by two reasons. First, cancer cell migration its highly dependent on cell–cell adhesion and cell–ECM mechanotransduction.^52^ Hence, as the LapNC supplemented matrix is stiffer, cells need to exert higher forces to move. Apart from that, LapNC hydrogels possessed more tortuous paths for migration, which reduced the movement of individual cells. Thus, softer networks facilitate cell migration. This outcome is in agreement with the mechanical characterization of the 2.5 mg/mL hydrogels: superior mechanical properties (*p* < 0.01, Figure 3) were shown for LapNC supplemented hydrogel, which also presented a highly reduced permeability (*p* < 0.005, Figure 4B).

To explore the effect of LapNC into the development of PACA multicellular clusters of cells in more detail, we chose these low stiffness hydrogels (2.5 mg/mL) of collagen type-I since they were previously characterized with/without LapNC. Then, we implemented fluorescence time-lapse microscopy with Lightsheet microscopy that does not compromise cell viability and is compatible with long-term fluorescent time-lapse. PACA nuclei were recorded from day 1 (single cells) up to day 3 (Figure 7D).

We observed that PACA cells cultures embedded in LapNC supplemented 2.5 mg/mL hydrogels induced a high degree of spheroid formation compared with their aforementioned controls without the clays (Figure 7B). This outcome is, indeed, related to the reduction in cell migration. Collecting all the outcomes from the low collagen concentration, we observed a coherent upshot: control 2.5 mg/mL hydrogel either did not allow the formation of spheroids or was not able to hold a 15 days incubation (Figures 5B and 6). However, with the LapNC addition, we observed formation of several small spheroids (Figure 5B orange and Figure 7D).

Overall, our work indicates that the use of LapNC in collagen-based hydrogels can alter its mechanical properties and microarchitecture improving and accelerating cellular tumor growth *in vitro*. In fact, the addition of LapNC increases the macroscopic stiffness of the hydrogel, also reducing its permeability. Therefore, its use provides earlier and larger cellular tumor spheroids at hydrogels of 4 mg/mL supplemented with LapNC in all the tumor types analyzed.

### 3.5 Relevance of the Study and Other Considerations

Cellular spheroids can more closely mimic the *in vivo* cellular microenvironment compared to conventional 2D cell culture. Therefore, it has become a popular trend to utilize those multicellular structures as *in vitro* models for many tissue engineering research fields.^53,54^ For example, Patra et al. created spheroids from a cell suspension in one-channel microfludic devices composed of microwells (200 × 200 μm and 300 × 300 μm).^55^ They saw significant differences when applying anticancer drugs to the spheroids and 2D cultures, which also highlighted the relevance of 3D cancer cultures. Apart from that, other types of microfluidic devices are reported in the literature. For instance, other authors have created spheroids controlling the size (98–126 μm diameter) by a microfluidic droplet-based device.^56^

As tumor microenvironment plays a key role in cellular functions, 3D hydrogels are well suited to mimic *in vivo* ECM for cancer cell studies.^38^ These systems allow researchers to gain a greater understanding of the biology and drug sensitivities of various solid tumor types, including pancreatic, prostate, osteosarcoma, glioblastoma multiforme, lung, ovarian, or breast.^57,58^

Increased tissue stiffness is associated with pathologies, like cancer and fibrosis, among others. Thus, ECM stiffness is emerging as a mechanical cue that precedes disease and drives its progression by altering cellular behaviors.^59^ Targeting ECM mechanics, by preventing or reversing tissue stiffening or interrupting the cellular response, is a therapeutic approach with clinical potential. Consequently, being able to tailor and customize ECM stiffness could be a powerful method for personalized research and therapies in multiple fields of tissue engineering. This is an application that fits in this study, as we were able to modify the matrix properties and architecture without the need of increasing the collagen concentration.

Additionally, there are also many other emerging fields apart from cancer research, such as the enhancement of osteogenic differentiation of stem cells for bone tissue engineering. For instance, Xavier et al. embedded LapNC in gelatin methacrylate hydrogels and then photo-cross-linked to form a composite scaffold; which promoted ALP activity and mineralization in normal growth medium without any osteoconductive factors.^60^ As other innovative application, LapNC have been also investigated to supplement bioinks, either to adjust their mechanical properties and also as nanocarriers as gene delivery devices.^61,62^

In this work, we have focused on the potential of the collagen-based matrices to create 3D tumoral models by culturing tumor cell spheroids and the possibility to tailor matrix properties and architecture through LapNC addition. Despite the strong potential of this 3D model in studying cancer development and progression, it is essential to consider the model’s limitations and their impact on the conclusions drawn from the study. First, the thickness of the microfluidic devices is 300 μm, which creates a physical limit for the spheroid size. Although thicker devices can be fabricated with this technology, our aim is to understand how they start to be formed and how they self-organize depending on the microenvironmental conditions. Second, these spheroids were created from a single cell type, although different cell types could have been cocultured. Our spheroids grew from one single cell, which did not allow to grow large spheroids in short times, and we could not analyze the necrotic core characteristic of larger spheroids.

Moreover, the biological features of the spheroids were not determined here. In order to examine the functionality as cancer model, it would be necessary to conduct further experiments, such as hematoxylin and eosin staining, TEM or immunohistochemistry^63^ to observe, for instance, the inner structure of the cluster.^64^ Additionally, the variation of collagen concentration and the hydrogel supplementation with LapNC affect both stiffness and microarchitecture. Thus, modifying mechanical properties maintaining hydrogel microstructure was not feasible. Nonetheless, despite these limitations, our approach provides a methodology for studying the initiation of tumor spheroid formation.^65^

## 4 Conclusions

Based on our results, we can infer that incorporating LapNC into a collagen-based matrix offers a means to customize the mechanical properties of the hydrogel, regardless of the initial amount of collagen used. The presence of nanoclays resulted in noteworthy alterations in the biophysical properties, permeability, and swelling ratio of the hydrogels. We conducted spheroid growth tracking in microfludic devices to present a valuable application of this innovative collagen-based culture system. To evaluate the spheroid growth we used three tumoral cell lines finding that the largest spheroids formed in LapNC-supplemented 4 mg/mL collagen hydrogels. The data showed that a proper combination of collagen concentration and LapNC may allow controlling the spheroid growth over time. Put together, our findings suggests that the use of LapNC in collagen-based hydrogels can provide improved mechanical properties and microarchitecture for tumor growth applications, opening up new possibilities for cancer research and other areas.

## Data Availability

The data that support the findings of this study are available from the corresponding author upon reasonable request.

## Supporting Information Available

Results of experiments conducted with two additional cell lines (PANC-1 and A549), a table that summarizes the statistics of the three cell lines, and finally, a figure that visually and schematically represents how we were able to quantify the parameters using a Matlab program (PDF)
