# Supplementary material for: Collagen-Laponite Nanoclay Hydrogels for Tumor Spheroid Growth
Source: Biomacromolecules. 2023 May 30;24(6):2879–91. doi: 10.1021/acs.biomac.3c00257 (PMC10265657; doi:10.1021/acs.biomac.3c00257)
Supplement: Supplementary file 1 — bm3c00257_si_001.pdf [file bm3c00257_si_001.pdf]

Collagen-laponite nanoclay hydrogels for tumor spheroid growth

Supplementary material

Pilar Alamán-Díez [a], Carlos Borau [a], Pedro Enrique Guerrero [a], Hippolyte Amaveda [b], Mario Mora [b], José María Fraile [c], Elena García-Gareta [a,d], José Manuel García Aznar [a], María Ángeles Pérez [a]

[a]*Multiscale in Mechanical and Biological Engineering, Aragón Institute of Engineering Research (I3A) & Aragón Institute of Healthcare Research (IIS Aragón), University of Zaragoza, Zaragoza, Spain,*

[b]*Aragon Institute of Nanoscience and Materials (INMA), University of Zaragoza & CSIC, Zaragoza, Aragon, Spain,*

[c]*Institute of Chemical synthesis and Homogeneous Catalysis (ISQCH), University ofZaragoza & CSIC, Zaragoza, Aragon, Spain.,*

[d]*Division of Biomaterials and Tissue Engineering, UCL Eastman Dental Institute, University College London, London, UK.*

Table S 1 – Spheroids area in μm² at the end of the culture in the different matrices. Median and p90 values are shown, corresponding to the final value of the Figure 6.

	Value	2C	2L	4C	4L	6C	6L
**PACA**	*Median*	-	5459.56	9890.79	10136.2	6866.35	4846.45
	P90	-	33898.57	21131.92	58804.07	16108.78	14888.62
**PANC-1**	*Median*	-	-	13417	33475.3	11573.3	6412.2
	P90	-	-	38426.47	60496.54	37109.43	34286.33
**A549**	*Median*	-	-	6061.85	7874.13	3977.08	5142.73
	P90	-	-	15164.36	20577.42	8183.82	11073.53

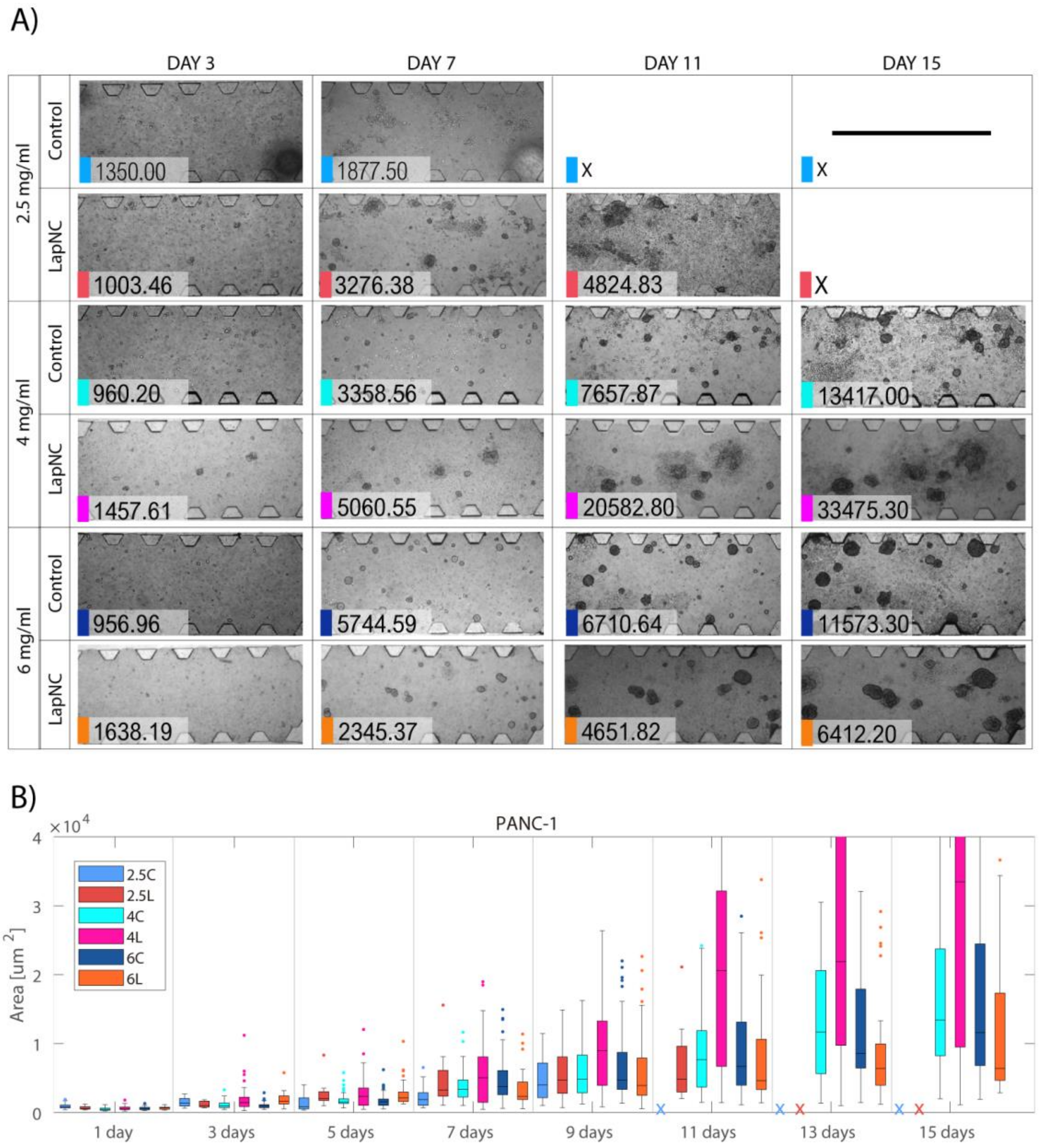

Figure S 1 – A) Brightfield images of a representative PANC-1 culture sample of each condition (collagen concentrations with/without LapNC) at 4 different timepoints. Each representative image is accompanied by the spheroid area median (µm^2) in that condition}. The symbol X denotes that the hydrogel collapsed at this timepoint or that no cells remained in 3D in this condition (total migration to the bottom surface). Scale bar in black 2 mm. B) PACA cells spheroid area (µm^2) distribution over time: 3D cultures in different matrix: 2.5, 4 and 6 mg/ml of collagen; with and without the addition of LapNC. Conditions not able to form spheroids at specific time points are marked with coloured crosses.

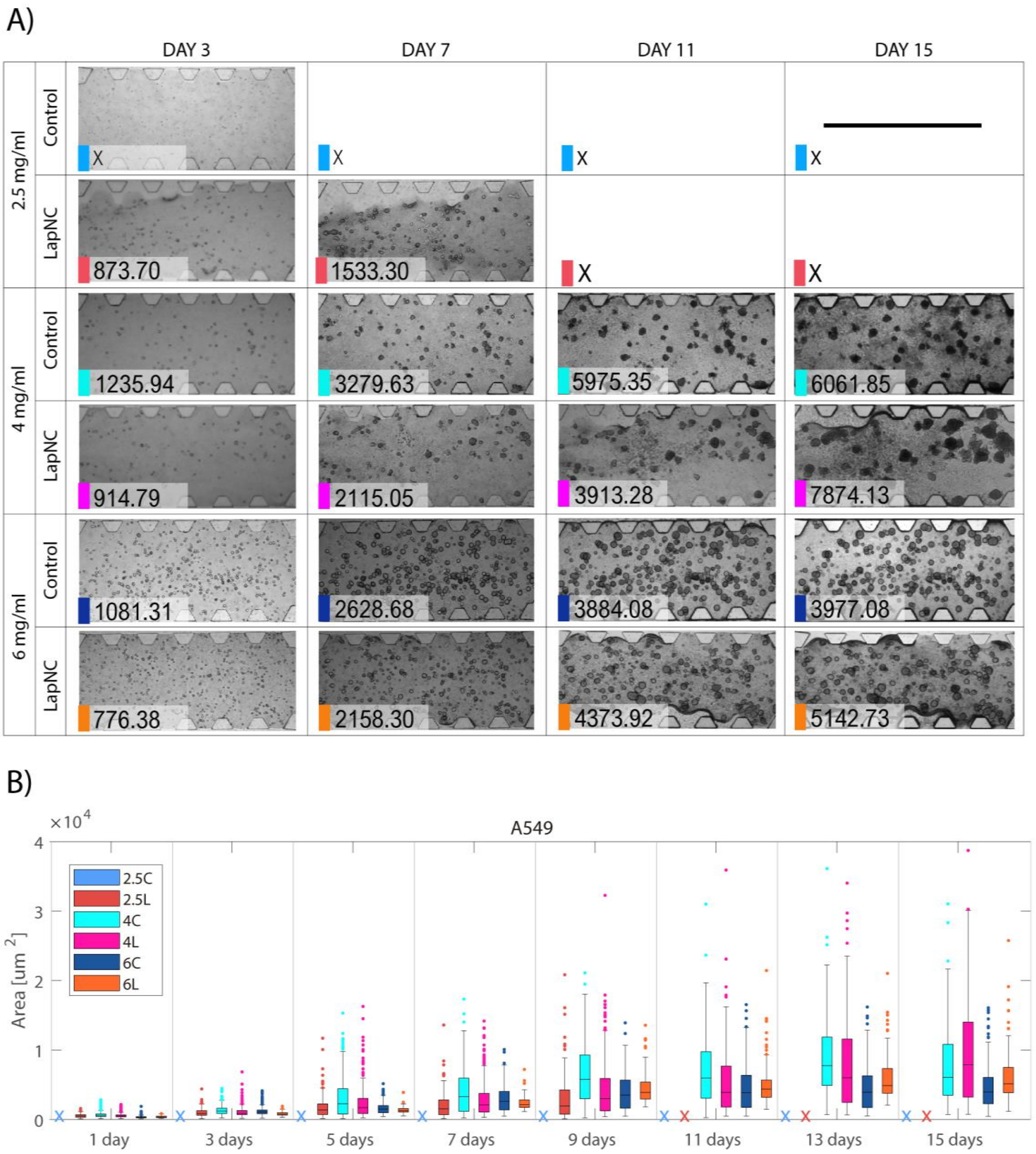

Figure S 2 – A) Brightfield images of a representative A549 culture sample of each condition (collagen concentrations with/without LapNC) at 4 different timepoints. Each representative image is accompanied by the spheroid area median (μm^2) in that condition}. The symbol X denotes that the hydrogel collapsed at this timepoint or that no cells remained in 3D in this condition (total migration to the bottom surface). Scale bar in black 2 mm. B) PACA cells spheroid area (μm^2) distribution over time: 3D cultures in different matrix: 2.5, 4 and 6 mg/ml of collagen; with and without the addition of LapNC. Conditions not able to form spheroids at specific time points are marked with coloured crosses.

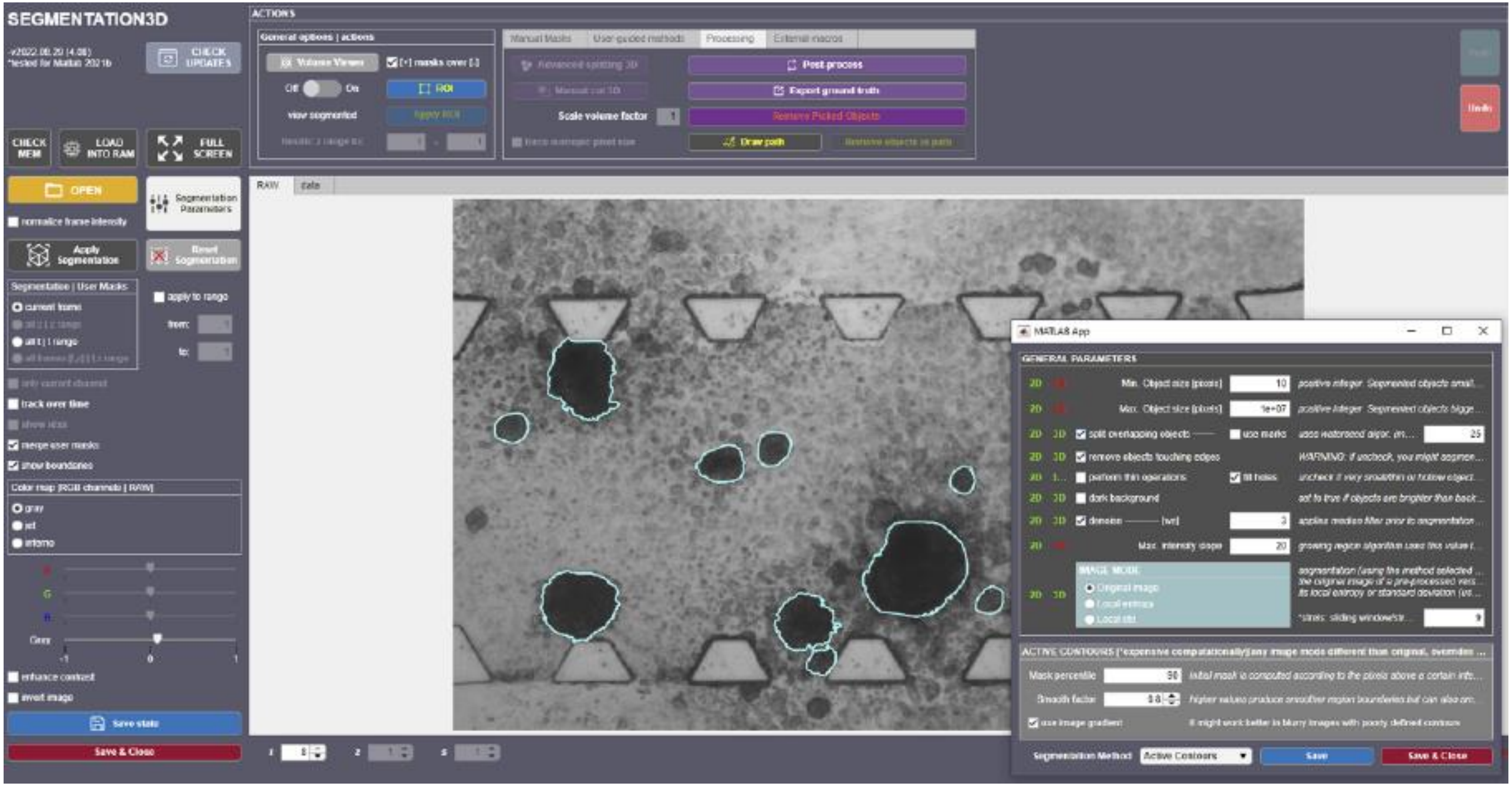

Figure S 3 – Example of the Matlab custom app interface used for automatic spheroid segmentation
